# Insights into the Control and Management of Human and Bovine African Trypanosomiasis in Zambia between 2009 and 2019—A Review

**DOI:** 10.3390/tropicalmed5030115

**Published:** 2020-07-11

**Authors:** Gloria M. Mulenga, Lars Henning, Kalinga Chilongo, Chrisborn Mubamba, Boniface Namangala, Bruce Gummow

**Affiliations:** 1 Department of Veterinary Services, Kakumbi Tsetse and Trypanosomiasis Research Station, P.O Box 70, Mfuwe 10101, Zambia; 2 Ministry of Fisheries and Livestock, Department of Veterinary Services, Lusaka 10101, Zambia; kchilongo@yahoo.co.uk (K.C.); chrisbornmw@yahoo.com (C.M.); 3 College of Public Health Medical and Veterinary Services, James Cook University, Townsville, Queensland 4814, Australia; lars.hening@jcu.edu.au (L.H.); bruce.gummow@jcu.edu.au (B.G.); 4 Institute of distance learning, The University of Zambia, Lusaka 10101, Zambia; b.namangala@unza.zm; 5 Faculty of Veterinary Science, University of Pretoria, 0028 Pretoria, South Africa

**Keywords:** trypanosomiasis, control, management, One Health, Zambia

## Abstract

Tsetse transmitted trypanosomiasis is a fatal disease commonly known as *Nagana* in cattle and sleeping sickness in humans. The disease threatens food security and has severe economic impact in Africa including most parts of Zambia. The level of effectiveness of commonly used African trypanosomiasis control methods has been reported in several studies. However, there have been no review studies on African trypanosomiasis control and management conducted in the context of One Health. This paper therefore seeks to fill this knowledge gap. A review of studies that have been conducted on African trypanosomiasis in Zambia between 2009 and 2019, with a focus on the control and management of trypanosomiasis was conducted. A total of 2238 articles were screened, with application of the search engines PubMed, PubMed Central and One Search. Out of these articles, 18 matched the required criteria and constituted the basis for the paper. An in-depth analysis of the 18 articles was conducted to identify knowledge gaps and evidence for best practices. Findings from this review provide stakeholders and health workers with a basis for prioritisation of African trypanosomiasis as an important neglected disease in Zambia and for formulation of One Health strategies for better control and/or management of the disease.

## 1. Introduction

African trypanosomiasis is endemic to Sub-Saharan Africa and continues to threaten human health and food security. African trypanosomiasis has been a major draw-back to agriculture and economic development in affected countries, with annual losses in agricultural gross domestic product estimated at USD 4.7 billion [1,2]. The current strategy of the Zambian government to preserve natural resources and create state protected National Parks (NPs) and Game Management Areas (GMAs) has led to an expansion of wildlife populations that serve as long term reservoirs for African trypanosomiasis, and also to an increase in the population of tsetse flies that transmit the disease [3]. At the same time, increase in human population density and the changing climate, particularly rainfall patterns, have forced people (and their livestock) to migrate into these GMAs in search of fertile land for farming. Such uncontrolled migration of people into protected areas has brought about changes in land use patterns that threaten to alter tsetse habitat quality and patterns of African trypanosomiasis transmission due to increased tsetse–human and tsetse–livestock contacts [4,5,6].

Tourists visiting NPs and GMAs have not been spared from risks of Human African Trypanosomiasis (HAT) infections occurring through transmission from wildlife reservoir hosts [6]. Despite cases of HAT reported from tourists after their visit to Zambia’s NPs, [7,8] there are gaps in protecting tourists and international travellers from tsetse and HAT. Some tour operators have taken it upon themselves to undertake some interventions, particularly in the form of tsetse control, aimed at reducing the risk of HAT infection among tourists visiting their facilities. Such limited interventions produce very limited levels of effectiveness or success, considering that such interventions need to cover considerable large proportions of the affected areas and as such require the collective input of many key stakeholders [9].

Tsetse flies are found in about 37% of Zambia’s land area, and it is estimated that the prevalence of African animal trypanosomiasis (AAT) in cattle ranges from 1% to 90% depending on the area [8]. Most of the affected areas are located in rural remote parts of the country and as such the direct negative impacts of the trypanosomiasis problem occur in communities that live in these areas. These impacts include serious economic consequences such as reduced livestock productivity and mortality and the high cost of treating affected livestock [10,11].

In Zambia, AAT has been managed through constant use of trypanocides by individual livestock farmers, while treatment and/or management of the disease in humans has been negatively affected by several factors that include late case detection that tends to result in tragic consequences (death) associated with adverse effects of the administered drugs in the late stage [12,13]. The Zambian government has generally made some notable strides in the control of African trypanosomiasis particularly through tsetse control. However, the government’s inability to put in place active surveillance systems, and the lack of adequate resources to effectively sustain control efforts, have contributed to the limitation of success associated with tsetse re-invasion and resurgence of African trypanosomiasis in areas where the disease had earlier been brought under control. In the case of HAT, lack of active surveillance systems has historically hindered progress towards the goal of eliminating African trypanosomiasis as a public health problem in Zambia [14,15].

The period between 2009 and 2019 has seen a significant number of undertakings focused largely on the parasite, transmission and epidemiology of African trypanosomiasis. However, no systematic review of the literature has been conducted on the control and management of African trypanosomiasis in Zambia particularly from a One Health perspective. This review seeks to address this knowledge gap.

## 2. Materials and Methods

**Figure A1:**
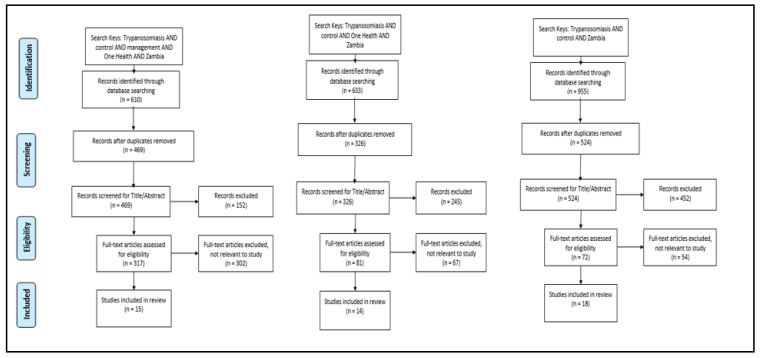
PRISMA diagrams showing three categories of searches conducted and selection criteria of literature included in the review.

With a focus on studies conducted on HAT and AAT control in Zambia, a systematic review (Figure A1 in Appendix A) of published data was undertaken. Using three searches with three categories of key words, a cumulative total of 2238 peer reviewed articles were identified in December 2019 from the following three search engines: PubMed, PubMed Central and One Search. One Search was used because it has a wider research area while PubMed was used because it is more aligned with veterinary sciences. Using the following key words: trypanosomiasis AND control AND management AND One Health AND Zambia, 610 articles were identified. In addition, two independent searches were performed using key words: trypanosomiasis AND control AND Zambia (995 articles identified), trypanosomiasis AND control AND One Health AND Zambia (633 articles identified). Duplicate articles were removed after which remaining articles were screened by title and abstract to assess the relevance of documents. Articles related to biochemical and biological developments in tsetse and African trypanosomiasis diagnostic assays were excluded from the review as most of the articles were focused on the trypanosome agent rather than management and control. Inclusion criteria were as follows: (i) studies conducted on the control and management of African trypanosomiasis in Zambia, (ii) related to One Health, (iii) related to African trypanosomiasis diagnostic methods, (iv) published in English only, and (v) published between January 2009 and December 2019. A final full text screening from the search conducted left 18 articles that met the inclusion criteria for the review (Table A1 in Appendix B). To support and supplement data from articles included in the review, published and unpublished government records and reports related to tsetse and African trypanosomiasis control for the same period were also referenced.

This review was conducted as part of a PhD project with ethical clearances from James Cook University (H7226 and A2498), Zambian Ethics Committee (Ref. No. 2018-Oct-001) and research approval from the Zambia National Health Research Authority.

## 3. Results

Based on the analysis of publications included in this review (Table A1 in Appendix B), results indicate that various trypanosome species circulate within a wide and diverse host community in Zambia [4]. The presence of the tsetse fly has facilitated the circulation of the parasite in the ecosystem [16]. Movement of people has led to the development of a new wildlife/livestock/human interface [4,17] *T. congolense* and *T. vivax* are the major causes of clinical AAT in cattle with low packed cell volume (PCV) usually an indicator of infection [18,19,20]. Infections with *T.b.r* in domestic animals remained a significant indicator that domestic animals could be reservoirs of HAT. Findings show that the impact of AAT is highest in cattle with dogs becoming a potential reservoir host for the human disease [16,21].

Current diagnostic methods used in Zambia do not conform to what is now thought to be the best practice [18,22,23]. Diagnosis of African trypanosomiasis remains a challenge in endemic areas of Zambia due to low staffing levels and non-functional laboratories [24,25].

Food security for communities living in tsetse-infested areas has continued to be negatively impacted [26]. The impact of AAT can be reduced through use of trypanocides and application of insecticide to control tsetse flies. Cattle farmers living in African trypanosomiasis-endemic areas and GMAs have resorted to drastic use of trypanocides to combat the disease [11]. African trypanosomiasis control in Zambia has been focused on cattle and not humans [26], with nothing published on the control and management of the disease in other domestic animals. Wildlife trypanosomiasis hosts pose a risk to communities and tourists living near or in national parks and game reserves [2,7,8].

Despite Zambia having had three major African trypanosomiasis control programmes (aerial spraying, insecticide treated targets and trypanocide drug use), the country has recorded several disease re-occurrences in areas where control was once undertaken. New cases are being reported in new areas while some old foci are disappearing [27,28]. Despite the evidence of the occurrence of African trypanosomiasis in both humans and livestock and the challenges faced by communities living in tsetse-infested areas, there is no One Health approach to control the disease [2,16,26].

A weak health system is in place for the management of HAT. Knowledge of HAT management among health workers is unsatisfactory [24]. A wide diversity of control programmes are available but lack government support [15,24,25,28]. Stakeholders in Zambia have competing views and beliefs regarding tsetse and African trypanosomiasis control, which is critical in developing a One Health approach for the control in both HAT and AAT. Environmentalists believe tsetse flies help keep environments wild and natural by stopping farmers encroaching protected areas. Agriculturalists feel that such moves have contributed to increased poverty as farmers are kept away from protected areas that are tsetse-infested [26].

## 4. Discussion

The Luangwa and Zambezi river basins support high densities of tsetse flies and wildlife reservoirs of African trypanosomiasis [4]. This review of tsetse and African trypanosomiasis studies undertaken in Zambia clearly indicates that most of these studies have been undertaken from or along the peripherals of the two river basins. With an estimated 37% of Zambia’s land area tsetse-infested, the risk of African trypanosomiasis infection for people and livestock living in the tsetse-infested areas in the country cannot therefore be overemphasised [11].

An assessment by the World Health Organization (WHO) indicated that HAT usually affected people whose occupations took them into tsetse-infested areas. Categories of people so affected include among others: small scale farmers, workers under wildlife services, tsetse control workers, poachers, honey gatherers and fishermen [13]. Increased human populations and thus increased demand for land for agriculture continues to force people and their livestock into tsetse-infested areas in search for fertile land. Migration of people with their livestock into tsetse-infested areas, as highlighted in this review, has resulted in changes in the epidemiology of African trypanosomiasis. Livestock rearing in these tsetse-infested areas has thus eroded the diverse ecosystems and led to the development of a new kind of wildlife/livestock/human interface with domestic animals acting as potential link for trypanosome exchange [4,14,29].

The risk of HAT infection in travellers to national parks and game reserves has however not received much attention. Despite reported cases of HAT from tourists after visiting tsetse-infested areas [7,8], there are currently no deliberate interventions in place to protect international travellers from tsetse flies and HAT. In Zambia, most tsetse interventions have been focused in areas with potential for livestock production, with little synchronisation with human intervention programmes [30,31]

Currently, African trypanosomiasis control in humans relies on early diagnosis and treatment. However, challenges in HAT diagnosis in rural settings of Zambia has hindered progress to the control of the disease. Most diagnostic health centres in rural Zambia depend on microscopy for diagnosis. Despite the low sensitivity associated with microscopy, the test remains the gold standard for both HAT and AAT diagnosis because it is affordable. However, the low sensitivity exhibited by microscopy makes it difficult to determine disease incidences, especially in cases where parasitaemia is low, thus stressing the need to improve field diagnosis of African trypanosomiasis [16,19,22,30].

Recent developments of molecular tools such as polymerase chain reaction (PCR) and loop-mediated isothermal amplification (LAMP) for detecting trypanosomiasis has provided hope for improving field diagnosis which may lead to eliminating African trypanosomiasis [32,33,34]. LAMP has been proven to be more sensitive than microscopy in detecting infections of *T. brucei* and *T. vivax* as compared to *T. congolense.* Such findings indicate the importance of LAMP in epidemiological studies related to HAT rather than AAT. The simplicity and sensitivity of LAMP makes it an ideal diagnostic tool for HAT [16,23,30]. On the other hand, multispecies PCR can identify several species of trypanosomes in a single PCR reaction, thus reducing the cost of molecular diagnosis. The main advantage of molecular tools over microscopy is for epidemiological studies and to identify different trypanosome species [35,36,37] other than point of care diagnostic tools. Limited support from relevant authorities has negatively impacted on the use of molecular methods in Zambia. Most molecular laboratory consumables cannot be sourced locally, therefore, procurement of consumables has remained a challenge even for institutions that have implemented the use of molecular tools.

For continued efforts to control African trypanosomiasis infections, there is a need to establish strong active and passive surveillance systems in African trypanosomiasis focal point areas. In the absence of diagnostic centres as seen in most rural settings of Zambia, departments of Health and Veterinary services can share resources, diagnostic capacities and personnel for improved case detection, treatment and control of African trypanosomiasis and other zoonotic diseases. Future control efforts for HAT may also consider simultaneous control of the disease in livestock and wildlife reservoirs as a One Health approach [25,26].

Meanwhile, lack of political commitment to sustain tsetse and African trypanosomiasis control programmes [38] has pushed livestock farmers to constant use of trypanocides. The study conducted by Mbewe et al. [12] confirms that livestock farmers living in GMAs or near NPs where tsetse challenge is high have resorted to constant trypanocide use to protect their livestock, which may have serious consequences related to trypanosome resistance to trypanocides [39]. Treatment of infected animals may seem to be the best option for most livestock farmers, but it may tend to be unsustainable and costly in the long run as AAT is largely a herd health problem (Tsetse and Trypanosomiasis section strategic plan 2020, Zambia-unpublished government record). Unfortunately, most farmers living in tsetse-infested areas treat their animals based on clinical signs and symptoms due to lack of access to laboratories and regular surveys from local veterinarians. In this case, most infections remain in their livestock populations and may be responsible for sustaining sporadic African trypanosomiasis incidences within their communities [40].

Earlier studies by Simukoko et al. [18], indicate that livestock treatment with trypanocides is dependent on seasonal variations of tsetse populations and the risk of AAT infection. Such findings indicate the need for tsetse and AAT control programmes to be focused on seasonal differences in the risk of AAT infection when tsetse challenge is highest. Key stakeholders can therefore use such findings to link to biological characteristics of the tsetse vector in developing cost effective and sustainable control programmes during periods of highest challenge [18,41]. From a travel medicine perspective, such findings also highlight risk periods for travellers.

Increased focus on communicable and non-communicable disease management has pushed African trypanosomiasis off the government’s priority list. There is a need to holistically quantify the impact and cost of African trypanosomiasis again in the context of disease prioritisation within Zambia and similarly affected countries. Lack of sustainable control programmes and the absence of a national surveillance and control programme for African trypanosomiasis among others, have impacted negatively on control efforts [24,25,26,38]. Breaking down barriers between social and natural scientists will help in developing a more holistic One Health approach to control tsetse flies and African trypanosomiasis in Zambia. Lessons learnt from past tsetse and African trypanosomiasis control operations can be useful in developing future cost effective and sustainable control programmes as well as informing health practitioners as to the risks travellers face in visiting these travel destinations and the in-country health support system available to them.

## 5. Recommendations

It is recommended that:

- Work is done to evaluate and identify African trypanosomiasis control programmes that are cost effective and sustainable in the regions where they are applied.
- Data on biological characteristics of tsetse and seasonal differences in African trypanosomiasis infection risk be considered when developing tsetse and trypanosomiasis control programmes in Zambia.
- More robust field diagnostic procedures for African trypanosomiasis be developed that consider the environmental, capacity and infrastructure constraints of working in countries like Zambia.
- Line Ministries consider sharing resources in order to improve diagnosis and treatment of African trypanosomiasis and other zoonotic diseases.
- A One Health approach be considered for the control of African trypanosomiasis in humans, livestock, wildlife and tsetse flies.

## Abbreviations

PCV	Packed Cell Volume
HAT	Human African Trypanosomiasis
PCR	Polymerase Chain Reaction;
LAMP	Loop Mediated Isothermal Amplification;
WBC	White Blood Cell;
CSF	Cerebrospinal Fluid;
GMA	Game Management Area;
CAT	Canine Animal Trypanosomiasis;
SRA	human Serum Resistance Associated;
RIME	Repetitive Insertion Mobile Element;
CON2-LAMP	specific primer targeting the 18Rrna gene of *T. congolense*; RHC, Rural Health Centre;
DNA	Deoxyribonucleic acid;
FAO	Food and Agriculture Organization RTTCP, Regional Tsetse and Trypanosomiasis Control Programme;
SAS/SAT	Sequential Aerial Spraying;
ITT	Insecticide Treated Targets and Traps;
TRY	trypanocidal drugs;
PATTEC	Pan African Tsetse and Trypanosomiasis Eradication Campaign;
AAT	Animal African Trypanosomiasis;
ITC	Insecticide Treated Cattle

## Appendix B

**Table A1:** Articles meeting selection criteria on trypanosomiasis control in Zambia between January 2009 and December 2019 and a summary of key findings.

Author Year	Study Aim	Study Design	Sample and Participation	Study Findings	Needs Domain
(Simukoko et al., 2011 [16]	To assess the monthly risk of bovine trypanosomiasis in cattle kept in tsetse-infested eastern plateau of Zambia.	Longitudinal study of bovine trypanosomiasis incidence in cattle	Eighty-five herds of cattle that grazed together were selected for a 19-month follow-up study	-The risk of trypanosome infection varied significantly between months with the higher risk recorded between December and February. -PCVs of infected and un-infected cattle did not differ significantly -*Trypanosoma congolense* and *T. vivax* were detected in 92.3% and 4.5% of the infected cattle, respectively. Mixed infections were detected in 3.2% of positive samples. -Overall, 155 infections were detected using PCR while microscopy detected 85 infections.	More effort in optimizing Animal African Trypanosomiasis (AAT) control during periods of highest challenges. Accuracy of AAT incidence using parasitological diagnosis stresses need for more sensitive diagnostic tools to improve field diagnosis.
(Mwanakasale and Songolo, 2011) [28]	-To identify districts in Zambia that were still reporting cases of Human African Trypanosomiasis (HAT). -To compare the occurrence of HAT cases before and after year 2000.	-Cross sectional survey of districts located close to national parks. -Literature review of occurrence of HAT in Zambia in the 1960s to 1990s.	-Conducted in nine provinces of Zambia except for Lusaka district. -Used google search, PubMed and world health organisation HINARI access to obtain data on HAT occurrence. Only articles with data on HAT distribution, epidemics, treatments and control of HAT before 2000 were reviewed.	-Chama, Mpika and Chipata districts were still reporting HAT cases. Seven districts that used to report HAT no longer had cases after January 2000. -All surveyed districts had no existing tsetse control programs. -In all surveyed health institutions, giemsa stain thick smear microscopy was the routine diagnostic method to detect HAT. Only Chilonga mission hospital used microhaematocrit centrifuge method to detect HAT. -Six of the surveyed hospitals had stocks of suramin but none had melarsoprol. -Findings from literature survey show a significant difference in HAT reporting foci from 1960s to 1990s and 2000 to 2007 with some old foci disappearing whilst new ones emerged or re-emerged	Districts reporting HAT 2008 to date Data on Agriculture practices between 2000 to 2007 and compared with 1960s to 1990s to confirm if agriculture practices may have contributed to reduced tsetse flies in previously tsetse-infested areas and thus the drastic reduction of HAT cases. Current data on human activities occurring in game management areas (GMAs) as they may be responsible for persistent HAT transmission and tsetse-human contacts. Human animal contacts as animals may carry trypanosomes with them. Poaching as game destruction was once used to eliminate wildlife reservoirs. Under diagnosing of HAT due to increased focus on management of HIV/AIDS and malaria.
(Anderson et al., 2011) [4]	To characterise the nature of the reservoir community for trypanosomiasis in the absence of influence from domesticated hosts	A cross-sectional survey of trypanosome prevalence in wildlife hosts. Conducted in the Luangwa valley from 2005 to 2007	A total of 418 wild animals were examined for the presence of trypanosomes	-Overall prevalence in all species was 13.9% with infection likely to be detected in waterbuck, lion, kudu and bushbuck, respectively. -Bushbuck indicated to be important hosts for *T. brucei s.l* with bushbuck, greater Kudu, and Lion to be important hosts for *T. congolense* while *T. vivax* was frequently detected in waterbuck. *-T. b. rhodesiense* were first identified in African buffalo and *T. brucei s.l* in leopard -First use multispecies PCR for the diagnosis of samples collected from free ranging wildlife which offers improved diagnostic specificity and sensitivity compared to traditional techniques. -Results indicated the ability of trypanosomes to survive in a wide variety of wildlife hosts.	Tsetse blood meal preference was identified as a risk factor for trypanosome infection. Difficulties in sampling wildlife and method used to sample in this study limited ability to investigate age as a risk factor in trypanosome infection Infection of *T. b. rhodesiense* in buffalo raises concern on possibility of infection been established in cattle populations not far from sampling area i.e., Mambwe district of the eastern province of Zambia. This is because buffalos move over large distances with potential to disseminate infection to other species. Trypanosome reservoir in wildlife hosts maybe wider that estimated in this study Influx of people with their livestock and land use may have an impact on the epidemiology of African trypanosomiasis.
(Namangala et al., 2012) [21]	To evaluate the performance of repetitive insertion mobile element (RIME)– loop mediated isothermal amplification (LAMP) and human serum resistance associated (SRA)–LAMP against microscopy in HAT diagnosis	Case study	Four male patients from Luangwa and Zambezi river basins	-Both RIME-LAMP and SRA-LAMP were able to detect *T. b. rhodesiense* in patients’ blood and in cerebrospinal fluid (CSF). -LAMP results correlated with microscopy results but they do not confirm the standard staging criteria using microscopy and white blood cell (WBC) in CSF.	Need for a detailed study with larger sample size to evaluate potential of LAMP to be used as a bedside diagnostic test for HAT and for making therapeutic decisions. Need for both active and passive surveillance of HAT and community sensitisation in HAT old foci.
(Namangala et al., 2013) [41]	To evaluate the performance of LAMP against microscopy to detect CAT in exotic dogs	Cross sectional survey of trypanosomiasis in exotic dogs	Six exotic dogs naturally infected with trypanosomes from Zambia’s South Luangwa National Park and Chiawa GMA.	-Results indicated first report of canine animal trypanosomiasis (CAT) in Zambia -All cases initially diagnosed by microscopy and later confirmed by LAMP, showing good correlation between the two methods. -Three dogs reported infection with *T. congolense* according to CON2-LAMP -All SRA-LAMP positive cases were also RIME-LAMP positive indicating similar sensitivity.	Further investigation on SRA gene isolated from two dogs in this communication. Scanty parasitaemia sometimes pose challenges caused by weak fluorescence signal thus need to quantify the fluorescence intensity and consider samples to be positive after subtracting the background fluorescence of the negative control. Dogs as potential source of HAT infections Need to investigate performance of LAMP in CAT diagnosis among locally bred dogs in tsetse-infested GMAs and National parks.
(Mwanakasale et al., 2013) [23]	To assess current health delivery system in the management of HAT.	Cross sectional survey of health institutions using structured questionnaires	Nine health institutions from Mpika district of Zambia were involved in the study	-The general knowledge on HAT of health staff from surveyed health institutions was unsatisfactory for proper management of the disease -Study revealed gross understaffing of essential staff to clinically diagnose and manage HAT-No staff from the surveyed institutions had received specific training on HAT diagnosis and treatment. -There was only one treatment centre (Chilonga mission hospital) from the surveyed health institutions -Erratic supply of trypanocides at the only treatment centre in the district -Only 2 of the surveyed institutions has functional laboratories with qualified personnel. Both institutions used less-sensitive methods to diagnose HAT -Distances between rural health centres (RHCs) and treatment centres and non-availability of transport to ferry suspected HAT patients.	Need for refresher courses to be conducted every two years for health personnel in districts at risk of HAT transmission in Zambia. Need for awareness on HAT for health policy makers so that they understand the need for refresher courses and trainings on disease management. Need to motivate in kind health staff at the frontline of identifying suspected cases and encourage them to refer such cases to diagnostic and treatment centres Need to establish Mpika district hospital as an additional treatment centre to decongest Chilonga mission hospital and improve health service delivery at both hospitals.Ministry of health to ensure that drugs for both stages of HAT are always in stock. Need for Ministry of health to equip and capacitate health institutions with laboratories and personnel as well as more sensitive diagnostic tools. Need for a proper referral system for HAT suspected cases to diagnostic treatment to ensure they reach their designated centres.
(Lisulo et al., 2014) [19]	To evaluate the performance of LAMP in determining trypanosome prevalence in indigenous dogs.	Cross sectional survey of Canine African Trypanosomiasis	A total of 237 indigenous dogs from 47 villages within five chiefdoms of Mambwe district of Zambia	-Fourteen cases of trypanosomes were detected using microscopy. -LAMP detected an additional 6 cases indicating higher sensitivity and specificity than microscopy. -Adult dogs were more likely to acquire CAT as they are involved in hunting. -CAT was significantly related to corneal opacity -Dogs are potential links for trypanosome exchange between livestock and humans.	Diagnostic accuracy of LAMP against microscopy suggested that its use in CAT diagnosis could improve disease management in African trypanosomiasis in endemic areas. Results from study can trigger a One Health approach towards control of HAT through disease intervention in livestock. Need for continuous surveillance of African trypanosomiasis in tsetse-infested regions using more user friendly and sensitive tests such as LAMP. Need to sensitise locals in GMAs potential dangers of keeping dogs that are left to scavenge without receiving veterinary services. Dogs may harbour other zoonoses apart from *T. b. rhodesiense* with potential serious implications to human health.
(Nyimba et al., 2015) [30]	To determine the prevalence and species distribution of caprine trypanosomiasis	Cross-sectional cluster survey of AAT in goats	Overall, 422 goats from Kalomo and Sinazongwe districts of Southern province of Zambia	-One goat was found infected on microscopy while 100 goats reported positive for AAT on LAMP. -Infection rate for Sinazongwe district was 22.4% while that for Kalomo district was 24.7% -*Trypanosoma brucei, T. vivax* and *T. congolense* were detected in 82.0%, 31.0% and 23.0% of the infected goats, respectively. Mixed infections were detected in 33.0% of positive samples. -Study results indicate the re-emergence of AAT in study areas were aerial spraying was once conducted by the government.	Need for improved staffing to enhance disease prevention and containment. Need for refresher courses for frontline Veterinary staff in order to improve service delivery. Need for sustainable control operations to avoid tsetse re-invasions and re-occurrence of disease in areas where control was once a success story.
(Laohasinnarong et al., 2015) [14]	To examine the presence of different trypanosome species in cattle, goats and tsetse using a combination of microscopy, PCR and LAMP	Cross sectional survey of trypanosomes in cattle, goats and tsetse flies.	In total, 243 cattle, 36 goats and 546 tsetse flies were examined for the presence of trypanosomes. Study conducted from Petauke, Chama and Isoka districts of Zambia.	-Microscopy exhibited relatively low sensitivity than PCR and LAMP -There was poor agreement among test methods. For instance, failure of PCR and LAMP to detect microscopically positive samples. -KIN PCR was found to be sensitive for detecting *T. congolense* -TviCatL-PCR and PFL-LAMP were better for detecting *T. Vivax* and *T. b. rhodesiense,* respectively. -The presence of *T. b. rhodesiense* in tsetse samples indicates its ability to take blood meal from multiple hosts (wildlife, humans and domestic animals), facilitating the circulation of the parasite in the ecosystem. -Infection in cattle and goats was highest with *T. congolense* and least with *T. vivax*	Need to establish if trypanosome DNA detected from cattle, goats and tsetse were active infections or residual DNA from dead trypanosomes picked from blood meals or treated animals. Need for a One Health approach towards the control of HAT through disease intervention in livestock, wildlife and tsetse.
(Mulenga et al., 2015) [22]	To investigate health personnel’s and health centre’s capacity to diagnose Human African trypanosomiasis	Cross sectional survey using structured questionnaires.	A sample of 101 health personnel drawn from 12 and nine health centres from Chama and Mambwe districts, respectively	-Staffing levels from both districts were extremely low with most health centres manned by one trained staff -Staff had basic knowledge to identify HAT with staff from Chama districts more likely to identify a case compared to their Mambwe counterparts. -Only Chama district had functional laboratories. Most health centres surveyed reported frequent use of rapid test kits for diagnosing mainly malaria parasites thus reducing diagnosis of other blood parasites that can be detected by microscopy including HAT.	Need for authorities to train and post more health staff in rural areas and to come up with deliberate policies that provide incentives to attract and motivate health workers in rural areas Need for capacity building and refresher trainings for health staff with regards to HAT diagnosis. Need for health centres located in HAT foci to be equipped with at least microscopes to enable them more easily identify cases when they occur. Further, referral or district hospitals can also be equipped with more sensitive laboratory tools like PCR and LAMP. Need for HAT national surveillance and control programmes to be enhanced.
(Mbewe et al., 2015b) [10]	To examine how socio- economic and environmental factors are associated with adherence to the recommended guidelines on trypanocide use	Cross sectional survey using a structured questionnaire.	Farmers interviewed from five veterinary camps from Itezhi tezhi district of central province of Zambia	-Of the interviewed farmers, 25.6% adhered to FAO guidelines on trypanocide use; (i) reducing the number of treatments on whole herd up to a maximum of four times in a year by integrating drug usage with other control measures and (ii) avoiding exposure of the whole parasite population to the drug by limiting treatments to individual sick animals. -None of the socio-economic factors (age, education, cattle herd size, competence in trypanocide use and access to extension on trypanocide use) were associated with a farmer’s adherence to FAO guidelines. -Low adherence to recommended FAO guidelines on trypanocide use was associated with the location of crush pen, whether in GMA or not, as an environmental factor. Farmers in GMAs were less likely to adhere to FAO guidelines than those in non-GMA.	Need for an integrated approach of measure to control AAT in the GMA of Itezhi tezhi to lessen overuse of trypanocides by farmers. Need to investigate if household income may influence farmer’s adherence to FAO guidelines of trypanocide use as defined in this study. Need to investigate if household income may influence control of vector borne diseases.
(Mbewe et al., 2015a) [20]	-To investigate the prevalence of animal trypanosomiasis in anaemic cattle	Cross sectional survey of AAT in cattle	A total of 564 Anaemic cattle from Itezhi tezhi district of Zambia	-Out of 564 cattle screened, 58 (10.3 %) had anaemia. PCR-RFLP results showed that 17 (29.3 %) anaemic cattle were positive for pathogenic trypanosomes compared to 1 (1.7 %) on parasitological examination using thick smears. -Infections were caused by *Trypanosoma congolense* and *Trypanosoma vivax*.	Need to investigate other anaemia causing factors in animal trypanosomiasis endemic areas of Itezhi tezhi district of Zambia.
(Grant et al., 2015) [24]	-To examine the narratives on African trypanosomiasis in Zambian policy. -To explore relationships between human, animal and environmental sectors	Case study of key informant interviews	Twenty participants from international organisations, research organisations and local activists.	-Environmentalists believed tsetse stop farmers encroaching protected areas thus keeping areas natural and wild. -Increased poverty because tsetse keeps farmers away from productive areas. -The Zambian government has other diseases of priority other than African trypanosomiasis and does not have funds to keep areas tsetse free. -Major focus of African trypanosomiasis control is emphasised on cattle and not humans. -The need to undertake tsetse control using the best methods have been identified but with no financial resources to support the plan. -Tsetse-infested forests that have been cleared for cotton growing have disrupted tsetse habitats due to chemicals used. -Current conservation strategies have sustained the preservation of tsetse flies and African trypanosomiasis.	Need for cross-sector, interdisciplinary decision making to stop rival narratives leading to competing actions. Need for a One Health approach to break down the barriers between social scientists, natural scientists and the expertise of the community.
(Mweempwa et al., 2015) [41]	To establish the impact of habitat fragmentation on the physiological and demographic parameters of tsetse flies in order to enhance the understanding of the relationship between fragmentation and AAT risk	Longitudinal study of tsetse age, abundance and trypanosome infection in areas of varying degrees of habitat fragmentation in eastern Zambia.	-A set of 3200 *Glossina morsitans morsitans* were caught using black screen fly rounds. -Overall, 577 female tsetse flies were dissected for ovarian age estimation. -A sentinel herd of 40 cattle was established at each of the four sites of Katete and Mambwe districts.	-Results indicated a significant increase in tsetse age as fragmentation increased. -Tsetse density was lower in most fragmented areas whilst the proportion of female flies increased significantly as fragmentation reduced. -AAT incidence in cattle was determined using buffy coat method. Infection rate in both cattle and tsetse flies was higher in highly fragmented areas.	Need to develop models that link biological characteristics of tsetse flies with habitat conditions. Such models maybe helpful in planning tsetse control interventions.
(Meyer et al., 2016) [25]	A literature review of past and on-going tsetse and African trypanosomiasis programmes	Systematic literature review of tsetse and African trypanosomiasis programmes between 1980 and 2015	Five African countries including Zambia. 68 documents plus 12 structured questionnaires reviewed.	-Twenty-three major Tsetse and Trypanosomiasis control programmes recorded from the five countries. Three control programmes conducted in Zambia during the stated period include the following: - Insecticide treated targets and traps (ITT) + trypanocidal drugs (TRY) in western province under government services for tsetse elimination (1987–1989). - Sequential aerial spraying (SAS) + ITT in eastern province under Regional Tsetse and Trypanosomiasis Control Programme (RTTCP) for tsetse control (1989–1994) -SAS + ITT in Kwando Zambezi belt under Pan African Tsetse and Trypanosomiasis Eradication Campaign (PATTEC) for tsetse elimination (2008 onwards)	Need for evaluation of the control programmes recorded. Need for standardised protocols to conduct such evaluations of control programmes
(Alderton et al., 2016) [15]	To develop an agent-based model (ABM) for investigating *Trypanosoma brucei rhodesiense*	-Mixed methods	-ABM comprised of human/animal trypanosomiasis and tsetse ecological survey data obtained along the 75km transect in the Luangwa valley of Zambia. -Ethnicity, age and gender data were also incorporated.	-ABM produced output that could not be readily generated by other techniques. On average there were 1.99 (S.E. 0.245) human infections and 1.83 (S.E. 0.183) cattle infections per 6-month period. -The model output identified that the approximate incidence rate (per 1000 person-years) was lower amongst cattle owning households (0.079, S.E. 0.017), than those without cattle (0.134, S.E. 0.017). - Immigrant tribes (e.g., Bemba I.R. = 0.353, S.E.0.155) and school-age children (e.g., 5–10-year-old I.R. = 0.239, S.E. 0.041) were the most at-risk for acquiring infection.	The ABM can be used as a tool for scenario testing at an appropriate spatial scale to allow the design of logistically feasible mitigation strategies suggested by model output. This is of importance where resources are limited, and management strategies are often pushed to the local scale.
(Holt et al., 2016) [2]	To assess AAT vulnerability in cattle owing communities	Cross sectional survey of cattle owners using questionnaire interviews.	210 households from Lundazi and Mambwe districts of Zambia	-AAT was constant with seasonal pattern, some trypano-tolerant breeds and communal grazing, small/moderate herd size with crops and mixing farming as primary income source, losses to draft reported, slightly higher mortalities and moderate costs diagnosing and treating, less likely to report treatment failure, low/good knowledge of control and tsetse traps/targets reported. -moderate AAT challenge, some concerns with resistance reported and most likely to keep pigs while some keep sheep and goats.	Need to integrate novel treatments with new and existing diagnostic and control programmes with findings of the study to develop tailored recommendations for AAT control and the reduce its impact in vulnerable communities.
(Meyer et al., 2018) [27]	To propose a framework for conducting a cost benefit analysis of possible AAT control analysis	A literature review of AAT of cattle production, herd management, impact of AAT on productivity, incidence and mortality	Two districts from Cameroon and Zambia (Mambwe district)	-For Zambia, the 10-year impact of tsetse elimination on the net value of cattle production was calculated as benefit–cost ratios using a discount rate of 5% and indicated the following:-2.3 (1.8–2.7) Targets, insecticide treated cattle (ITC) barrier -2.0 (1.6–2.4) Targets, barrier traps -2.8 (2.3–3.3) Aerial spraying, ITC barrier -2.5 (2.0–2.9) Aerial spraying, barrier trap -The use of SAT as elimination method for Mambwe district yielded a higher benefit–cost ratio than the use of targets. -The model estimated the total discounted control costs at 3.8 million USD and benefits at 10.5 million USD for Mambwe district if SAT was used as tsetse elimination method	Need for barriers to be maintained and monitoring activities conducted continuously unless sequential elimination of the entire tsetse belt is achieved. Cost–benefit studies should be supported by recent estimates of key parameters such as frequency of trypanosome infection and impact, livestock and tsetse demographics. Model generated in study combined data from different locations and from studies conducted years ago, there is need to validate the model using current data from same locations. Need to use existing control programmes for designing future control programmes.

## References

[1] Swallow B.M. (2000). Impacts of Trypanosomiasis on African Agriculture.

[2] Holt H.R., Selby R., Mumba C., Napier G.B., Guitian J. (2016). Assessment of Animal African Trypanosomiasis (AAT) Vulnerability in Cattle-Owning Communities of Sub-Saharan Africa. Parasites Vectors.

[3] Munang’andu H.M., Siamudaala V., Munyeme M., Nalubamba K.S. (2012). A review of ecological factors associated with the epidemiology of wildlife trypanosomiasis in the luangwa and zambezi valley ecosystems of zambia. Interdiscip Perspect Infect. Dis..

[4] Anderson N.E., Mubanga J., Fevre E.M., Picozzi K., Eisler M.C., Thomas R., Welburn S.C. (2011). Characterisation of the wildlife reservoir community for human and animal trypanosomiasis in the Luangwa Valley, Zambia. PLoS Negl. Trop. Dis..

[5] Anderson N.E., Mubanga J., Machila N., Atkinson P.M., Dzingirai V., Welburn S.C. (2015). Sleeping sickness and its relationship with development and biodiversity conservation in the Luangwa Valley, Zambia. Parasites Vectors.

[6] Muriuki G.W., Njoka T.J., Reid R.S., Nyariki D.M. (2005). Tsetse control and land-use change in Lambwe valley, south-western Kenya. Agric. Ecosyst. Environ..

[7] Frean J., Sieling W., Pahad H., Shoul E., Blumberg L. (2018). Clinical management of East. African trypanosomiasis in South. Africa: Lessons learned. Int. J. Infect. Dis..

[8] Richter J., Göbels S., Göbel T., Westenfeld R., Müller-Stöver I., Häussinger D. (2012). A returning traveller with fever, facial swelling, and skin lesions. BMJ.

[9] Kakumbi (2017). Annual Report.

[10] Mwiinde A.M., Simuunza M., Namangala B., Chama-Chiliba C.M., Machila N., Anderson N., Welburn S.C. (2017). Estimating the economic and social consequences for patients diagnosed with human African trypanosomiasis in Muchinga, Lusaka and Eastern Provinces of Zambia (2004–2014). Infect. Dis. Poverty.

[11] Livestock and Fisheries (2016). Ministry Annual Report.

[12] Mbewe N.J., Sitali L., Namangala B., Michelo C. (2015). Adherence to the Food and Agricultural Organization guidelines on trypanocide usage among cattle farmers in Itezhi tezhi, Central Zambia. Vet. Parasitol..

[13] WHO WHO Fact Sheet-Human African Trypanosomiais. https://www.who.int/news-room/fact-sheets/detail/trypanosomiasis-human-african-(sleeping-sickness).

[14] Franco J.R., Cecchi G., Priotto G., Paone M., Diarra A., Grout L., Argaw D. (2020). Monitoring the elimination of human African trypanosomiasis at continental and country level: Update to 2018. PLoS Negl. Trop. Dis..

[15] Mwanakasale V., Songolo P. (2011). Disapperance of some Human African Trypanosomiasis transmission foci in Zambia in the absence of tsetse fly and trypanosomiasis control program over a period of forty years. Trans. R. Soc. Trop. Med. Hyg..

[16] Laohasinnarong D., Goto Y., Asada M., Nakao R., Hayashida K., Kajino K., Namangala B. (2015). Studies of trypanosomiasis in the Luangwa valley, north-eastern Zambia. Parasit Vectors.

[17] Alderton S., Macleod E.T., Anderson N.E., Schaten K., Kuleszo J., Simuunza M., Atkinson P.M. (2016). A Multi-Host Agent-Based Model. for a Zoonotic, Vector-Borne Disease. A Case Study on Trypanosomiasis in Eastern Province, Zambia. PLoS Negl. Trop. Dis..

[18] Simukoko H., Marcotty T., Vercruysse J., Van den Bossche P. (2011). Bovine trypanosomiasis risk in an endemic area on the eastern plateau of Zambia. Res. Vet. Sci..

[19] Marcotty T., Simukoko H., Berkvens D., Vercruysse J., Praet N., Van den Bossche P. (2008). Evaluating the use of packed cell volume as an indicator of trypanosomal infections in cattle in eastern Zambia. Prev. Vet. Med..

[20] Mweempwa C., Marcotty T., De Pus C., Penzhorn B.L., Dicko A.H., Bouyer J., De Deken R. (2015). Impact of habitat fragmentation on tsetse populations and trypanosomiasis risk in eastern Zambia. Parasites Vectors.

[21] Lisulo M., Sugimoto C., Kajino K., Hayashida K., Mudenda M., Moonga L., Namangala B. (2014). Determination of the prevalence of African trypanosome species in indigenous dogs of Mambwe district, eastern Zambia, by loop-mediated isothermal amplification. Parasit Vectors.

[22] Mbewe N.J., Namangala B., Sitali L., Vorster I., Michelo C. (2015). Prevalence of pathogenic trypanosomes in anaemic cattle from trypanosomosis challenged areas of Itezhi-tezhi district in central Zambia. Parasit Vectors.

[23] Namangala B., Hachaambwa L., Kajino K., Mweene A.S., Hayashida K., Simuunza M., Moonga L. (2012). The use of Loop-mediated Isothermal Amplification (LAMP) to detect the re-emerging Human African Trypanosomiasis (HAT) in the Luangwa and Zambezi valleys. Parasit Vectors.

[24] Mulenga G.M., Likwa R.N., Namangala B. (2015). Assessing the capacity to diagnose human African trypanosomiasis among health care personnel from Chama and Mambwe districts of eastern Zambia. BMC Res. Notes.

[25] Mwanakasale V., Songolo P., Daka V. (2013). Challenges in the control of human African trypanosomiasis in the Mpika district of Zambia. BMC Res. Notes.

[26] Grant C., Anderson N., Machila N. (2015). Stakeholder Narratives on Trypanosomiasis, Their Effect on Policy and the Scope for One Health. PLoS Negl. Trop. Dis..

[27] Meyer A., Holt H.R., Selby R., Guitian J. (2016). Past and Ongoing Tsetse and Animal Trypanosomiasis Control. Operations in Five African Countries: A Systematic Review. PLoS Negl. Trop. Dis..

[28] Meyer A., Holt H.R., Oumarou F., Chilongo K., Gilbert W., Fauron A., Guitian J. (2018). Integrated cost-benefit analysis of tsetse control and herd productivity to inform control programs for animal African trypanosomiasis. Parasit Vectors.

[29] Squarre D., Kabongo I., Munyeme M., Mumba C., Mwasinga W., Hachaambwa L., Namangala B. (2016). Human African Trypanosomiasis in the Kafue National Park, Zambia. PLoS Negl. Trop. Dis..

[30] Nyimba P.H., Komba EV G., Sugimoto C., Namangala B. (2015). Prevalence and species distribution of caprine trypanosomosis in Sinazongwe and Kalomo districts of Zambia. Vet. Parasitol..

[31] Alderton S., Macleod E.T., Anderson N.E., Machila N., Simuunza M., Welburn S.C., Atkinson P.M. (2018). Exploring the effect of human and animal population growth on vector-borne disease transmission with an agent-based model of Rhodesian human African trypanosomiasis in eastern province, Zambia. PLoS Negl. Trop. Dis..

[32] Delespaux V., Dinka H., Masumu J., Van den Bossche P., Geerts S. (2008). Five-fold increase in Trypanosoma congolense isolates resistant to diminazene aceturate over a seven-year period in Eastern Zambia. Drug Resist. Updates.

[33] Njiru Z.K., Mikosza A.S.J., Matovu E., Enyaru J.C.K., Ouma J.O., Kibona S.N., Ndung’u J.M. (2008). African trypanosomiasis: Sensitive and rapid detection of the sub-genus Trypanozoon by loop-mediated isothermal amplification (LAMP) of parasite DNA. Int. J. Parasitol..

[34] Njiru Z. (2012). Loop-Mediated Isothermal Amplification Technology: Towards Point of Care Diagnostics. PLos Negl. Trop. Dis..

[35] Njiru Z.K., Constantine C.C., Guya S., Crowther J., Kiragu J.M., Thompson R.C.A., Dávila A.M.R. (2005). The use of ITS1 rDNA PCR in detecting pathogenic African trypanosomes. Parasitol. Res..

[36] Ahmed H.A., Picozzi K., Welburn S.C., MacLeod E.T. (2013). A comparative evaluation of PCR- based methods for species- specific determination of African animal trypanosomes in Ugandan cattle. Parasit Vectors.

[37] Picozzi K., Carrington M., Welburn S.C. (2008). A multiplex PCR that discriminates between Trypanosoma brucei brucei and zoonotic T. b. rhodesiense. Exp. Parasitol..

[38] Mulenga G.M., Namangala B., Likwa R.N. (2016). Capacity and Policy Change in Managing Trypanosomiasis in Endemic Rural Health Centres of Eastern Zambia. ARC J. Public Health Community Med..

[39] Van den Bossche P., Doran M., Connor R.J. (2000). An analysis of trypanocidal drug use in the Eastern province of Zambia. Acta Trop..

[40] Von Wissmann B., Machila N., Picozzi K., Fèvre E.M., Barend M., Handel I.G., Welburn S.C. (2011). Factors associated with acquisition of human infective and animal infective trypanosome infections in domestic livestock in Western Kenya. PLoS Negl. Trop. Dis..

[41] Namangala B., Oparaocha E., Kajino K., Hayashida K., Moonga L., Inoue N., Sugimoto C. (2013). Preliminary investigation of trypanosomosis in exotic dog breeds from Zambia’s Luangwa and Zambezi valleys using LAM. Am. J. Trop. Med. Hyg..

